# Lineage Differentiation Markers as a Proxy for Embryo Viability in Farm Ungulates

**DOI:** 10.3389/fvets.2021.680539

**Published:** 2021-06-15

**Authors:** Alba Pérez-Gómez, Leopoldo González-Brusi, Pablo Bermejo-Álvarez, Priscila Ramos-Ibeas

**Affiliations:** Department of Animal Reproduction, National Institute for Agriculture and Food Research and Technology (INIA), Madrid, Spain

**Keywords:** embryo quality, embryo transfer, developmental biology, lineage markers, viability, conceptus elongation, assisted reproductive technologies, post-hatching embryo culture

## Abstract

Embryonic losses constitute a major burden for reproductive efficiency of farm animals. Pregnancy losses in ungulate species, which include cattle, pigs, sheep and goats, majorly occur during the second week of gestation, when the embryo experiences a series of cell differentiation, proliferation, and migration processes encompassed under the term conceptus elongation. Conceptus elongation takes place following blastocyst hatching and involves a massive proliferation of the extraembryonic membranes trophoblast and hypoblast, and the formation of flat embryonic disc derived from the epiblast, which ultimately gastrulates generating the three germ layers. This process occurs prior to implantation and it is exclusive from ungulates, as embryos from other mammalian species such as rodents or humans implant right after hatching. The critical differences in embryo development between ungulates and mice, the most studied mammalian model, have precluded the identification of the genes governing lineage differentiation in livestock species. Furthermore, conceptus elongation has not been recapitulated *in vitro*, hindering the study of these cellular events. Luckily, recent advances on transcriptomics, genome modification and post-hatching *in vitro* culture are shedding light into this largely unknown developmental window, uncovering possible molecular markers to determine embryo quality. In this review, we summarize the events occurring during ungulate pre-implantation development, highlighting recent findings which reveal that several dogmas in Developmental Biology established by knock-out murine models do not hold true for other mammals, including humans and farm animals. The developmental failures associated to *in vitro* produced embryos in farm animals are also discussed together with Developmental Biology tools to assess embryo quality, including molecular markers to assess proper lineage commitment and a post-hatching *in vitro* culture system able to directly determine developmental potential circumventing the need of experimental animals.

## Introduction

Optimal reproductive performance in farm animals relies on the proper accomplishment of the different biological processes leading to delivery. Starting from the ovulation of a competent oocyte, conception requires a successful fertilization to produce a zygote, which marks the onset of preimplantation development. During preimplantation development, complex cell proliferation, differentiation and migration processes must be finely controlled to ensure embryo viability and subsequent embryo implantation. Following embryo implantation, a fetus will be developed, ultimately resulting in a newborn. When global reproductive failures in farm ungulates are dissected into these steps, preimplantation development (i.e., the period compromised between fertilization and implantation) clearly stands out as the most problematic. For instance, in the case of cattle, embryonic losses prior to day 16 (D16) post-insemination can rise up to 50% in high yielding dairy cows (1), whereas in pigs it has been estimated that one into five embryos dies before implantation (2).

Preimplantation development in ungulates can be divided into two periods. The first period spans from fertilization to blastocyst hatching, i.e., the release of the embryo from a glycoprotein protective shell termed zona pellucida. This pre-hatching period is common to all mammals and constitutes the whole preimplantation period in rodents and humans, where blastocysts implant right after hatching. In contrast, ungulates exhibit a second preimplantation period termed conceptus elongation. During conceptus elongation the ungulate blastocyst must undergo dramatic morphological changes that, in the case of cattle, convert a ~150 μm D7 blastocyst into a ~30 cm long D21 conceptus around implantation (3, 4). Reproductive failures occurring during preimplantation development can be originated in any of these periods, but developmental collapse during conceptus elongation is the main responsible for global reproductive failures in ungulates (5). To illustrate the magnitude of this problem in cattle farms, it has been estimated that one third of the viable D6 blastocysts fail to elongate and maintain pregnancy by D28 (6), and embryo mortality during early conceptus elongation (D7–D14) oscillates between 26 and 34% (4). In this perspective, the study of the cell differentiation, proliferation, and migration processes occurring during preimplantation development is crucial to understand conceptus collapse and, thereby, finding suitable markers to assess proper lineages development is key to improve reproductive efficiency in livestock ungulates.

The molecular basis of the developmental processes occurring during the first weeks of pregnancy in ungulates is only partially understood, mainly due to two technical limitations: the lack of an *in vitro* system able to recapitulate conceptus elongation, and the difficulties for performing loss-of-function studies in these species. Luckily, recent advances in *in vitro* culture of post-hatching blastocysts in cattle (7) and sheep (own unpublished data), together with the development of CRISPR-Cas9 technology to perform loss of function studies in livestock species are set to boost our knowledge on molecular markers for assessing proper embryo development.

In this review, we discuss the differences between ungulate embryo development and that of rodents and humans, highlighting the molecular markers involved in the first lineages differentiation events occurring in ungulates. We also revise different studies that have reported impaired lineages development in *in vitro-*produced embryos, and provide insights into the potential of lineages markers and post-hatching embryo culture systems to assess embryo quality in farm animals.

## Molecular Control of the First Cell Lineage Differentiations

Segregation of the first cell lineages in the embryo is critical for proper pregnancy establishment and fetus development. Unfortunately, comprehensive understanding of this process is only available in mice, which became a classical model in Developmental Biology due to its low maintenance cost, fast life cycle and, particularly, due to the well-developed techniques for genome modification in this species. Although the first stages of early embryo development are broadly conserved in mammals, increasing amount of research using novel technologies such as single cell transcriptomics or generation of knock-out embryos by CRISPR-Cas9 are revealing important differences in gene regulatory networks between rodent and non-rodent species.

### The First Decision: Inner Cell Mass vs. Trophectoderm

**Figure 1:**
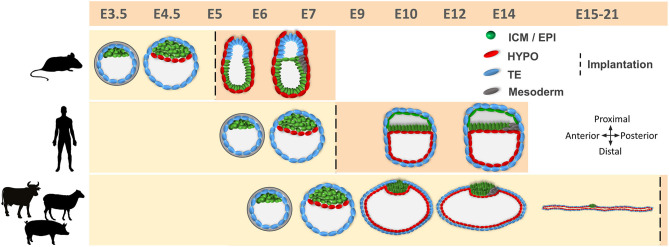
Comparative developmental timeline between mice, humans, and farm ungulates. Different cell lineages are indicated. Embryo development in humans and farm ungulates is delayed compared to mice. Implantation takes place after blastocyst hatching in mice and humans, while in farm ungulates it occurs following conceptus elongation. Mouse epiblast develops in a cup shape after the blastocyst stage, while in humans and farm ungulates it forms a flat embryonic disc. E, embryonic day; ICM, inner cell mass; EPI, epiblast; HYPO, hypoblast; TE, trophectoderm.

During the first cell divisions, the embryo relies on maternal transcripts and proteins until the embryonic genome is activated between the 2- (rodents) and 4/8-cell stages (lagomorphs, ungulates, and primates) (8). At these early stages of development, blastomeres are morphologically indistinguishable, but from the 8-cell stage in the mouse, cells located in the outside of the embryo undergo a process of polarization that will influence their fate, biasing outer polar cells toward trophectoderm (TE) and inner apolar cells toward inner cell mass (ICM) (9–11) (Figure 1). The formation of an apical domain in the outer cells triggers a transcriptional network involving Hippo/YAP signaling and the activation of *Tead4* in the mouse, which leads to the downregulation of the pluripotency factor *Sox2* and the upregulation of *Cdx2* from the morula stage (12–15). Activation of CDX2 downregulates *Oct4* (14, 16) and activates the expression of other TE markers such as *Gata3, Eomes* or *Elf5* (17), allowing the emergence of the first cell lineages during the formation of the blastocyst: TE, which will mediate implantation, and ICM that will form the embryo proper.

These transcription factors exhibit different temporal expression profiles and roles in cell differentiation events in non-rodent mammals (Table 1). TE-specific genes *CDX2, GATA3*, or *TEAD4* are also expressed in the TE of bovine (18–20), porcine (21, 22), and human (20, 23, 24) blastocysts, but remarkable differences in timing of expression and function have been observed between ungulates and rodents. For instance, CDX2 protein expression before the blastocyst stage is restricted to some scattered cells in pig and cattle, contrasting to the ubiquitous expression in mice (14, 25–27), and both CDX2 and OCT4 are expressed in the TE until late blastocyst stages in humans, pigs and cattle (25, 26, 28, 29), in contrast to the mutually exclusive expression observed in mouse blastocysts (14). In the same line, whereas CDX2 represses *Oct4* expression in the TE of murine blastocysts (16), *CDX2* downregulation does not affect *OCT4* expression in bovine embryos (25, 30), and bovine *OCT4* promoter lacks an essential region -CR4- necessary for repression of OCT4 in mouse TE (25). Recent KO experiments have also highlighted a different function of OCT4 in rodents vs. ungulates and humans. *Oct4* KO mouse embryos develop to blastocyst, although hypoblast formation—explained in the next section—is impaired, but the absence of OCT4 protein in *OCT4* KO morulae has been observed to impair blastocyst formation and reduce *CDX2* expression in both cattle (31) and humans (32, 33).

**Table 1:** Species-specific differences in the expression of lineage markers.

**Marker**		**Mouse**					**Human**					**Cow/pig**				
		**Early blastocyst**		**Late blastocyst**			**Early blastocyst**		**Late blastocyst**			**Early blastocyst**		**Late blastocyst**		
		**ICM**	**TE**	**EPI**	**HYPO**	**TE**	**ICM**	**TE**	**EPI**	**HYPO**	**TE**	**ICM**	**TE**	**EPI**	**HYPO**	**TE**
ICM/EPI	OCT4	+	–	+	–	–	+	+	+	+	+	+	+	+	+	+
	SOX2	+	–	+	–	–	+	–	+	–	–	+	–	+	–	–
	NANOG	+	–	+	–	–	+	–	+	–	–	+	–	+	–	–
HYPO	GATA6	+	–	–	+	–	+	+	–	+	–	+	+	–	+	–
	SOX17	+	–	–	+	–	+	–	–	+	–	+	–	–	+	–
	PDGFRA	+	–	–	+	–	+	–	–	+	–	+	–	–	+	–
	GATA4	+	–	–	+	–	+	–	–	+	–	+	–	–	+	–
TE	CDX2	–	+	–	–	+	–	+	–	–	+	–	+	–	–	+
	GATA3	–	+	–	–	+	–	+	–	–	+	–	+	–	–	+
	TEAD4	–	+	–	–	+	+	+	+	+	+	+	+	+	+	+
	EOMES	–	+	–	–	+	–	–	–	–	–	–	–	–	–	–
	ELF5	–	+	–	–	+	–	–	–	–	–	–	–	–	–	–

*Green (positive) and red (negative) colors indicate differences in the expression of specific markers between species*.

Other transcription factors involved in TE vs. ICM differentiation in mouse also seem to play different roles in ungulate development. TEAD4, the upstream regulator of TE genes such as *CDX2* and *EOMES*, does not appear to be essential for TE specification in ungulates, as its downregulation does not impair blastocyst formation in cattle (34, 35), whereas *Tead4* ablation in mice completely abolishes blastocoel formation (36, 37). Furthermore, transcription factors downstream *TEAD4* such as *EOMES* and *ELF5* are still not expressed at the blastocyst stage in cattle (18, 30, 38), pigs (21) and humans (24). Although gene ablation experiments in ungulates are required to faithfully elucidate the role of these genes, these evidences strongly suggest that the transcriptional network required for TE specification in ungulates is considerably different to that of mice. In this perspective, early TE specification could have emerged as an evolutionary mechanism in rodents to allow implantation at an earlier stage than in other species. Fortunately, recent improvements in genome edition techniques in ungulates (39, 40) currently allow the exploration of the molecular machinery involved in ICM/TE specification in farm animals. Genome editing constitutes also an unvaluable tool to study other reproductive processes (41). For instance, the generation of aromatase-null porcine conceptuses has uncovered that intrinsic estrogen conceptus production is not required for early maternal recognition of pregnancy or implantation in pig, in striking contrast to previous beliefs (42).

### The Second Decision: Epiblast vs. Hypoblast

After the differentiation of the TE, the second cell fate decision takes place in the ICM and determines the emergence of the pluripotent epiblast and the extraembryonic hypoblast (Figure 1). In mice, hypoblast markers are sequentially expressed, starting with GATA6 at the 8-cell, PDGFRα at the 16-cell, SOX17 at the 32-cell, and GATA4 at the 64-cell stages (43, 44). Later on, the cells forming the early ICM (E3.5) co-express both epiblast (OCT4, SOX2, and NANOG) and hypoblast (GATA6, SOX17, and PDGFRA) proteins, but by E4.5 these cells will show mutually exclusive expression for both markers (43, 45). Although the detailed temporal expression pattern of these markers is not available for most domestic mammals, there are broad similarities in the gene regulatory network controlling the second lineage specification within mammals (46). For example, *PDGFR*α and *SOX17* are also co-expressed with epiblast markers in the bipotent ICM cells and become restricted to hypoblast cells in late blastocysts in human, pig and cattle embryos (18, 21, 24). However, important differences have been also reported between rodent and non-rodent species (Table 1). GATA6, together with the epiblast marker NANOG, are expressed in all cells of the mouse morula and their mutual repression in the late ICM is essential for epiblast vs. hypoblast specification (43, 47). However, this might not be a strict requirement in all mammals, since GATA6 is expressed in all cell lineages of the blastocyst in primates, pigs and cattle, only becoming restricted to the hypoblast at later developmental stages (18, 21, 24, 48–50), and NANOG is not expressed in the human, pig and cattle morula (21, 26, 51, 52). Once specified, hypoblast cells reorganize to form an epithelium lying in contact with the blastocoel cavity (43) and migrate to cover the inner embryo surface in primates and ungulates (7) (Figure 1).

## Post-Hatching Development in Ungulates: Conceptus Elongation

A characteristic aspect of ungulate development is TE fate. After blastocyst hatching, the TE can be classified into mural TE, which covers the blastocoel cavity, and polar TE, covering the ICM. While in rodents and primates, the polar TE forms the extraembryonic ectoderm (ExE), contributing to implantation and becoming part of the placenta, in lagomorphs (53) and ungulates (3) this function is accomplished by the mural TE. The polar TE, also known as Rauber's layer (RL), is removed through an apoptotic mechanism (54) around D9–D11 in pigs (55), D10–D12 in horses (56), D11–D12 in sheep (own unpublished observations) and by day 14 in cattle (54), directly exposing the epiblast to the uterine histotroph (Figure 1). Shortly after the disappearance of the RL, the extraembryonic membranes (EEMs, composed by mural TE and hypoblast) undergo extensive proliferation. As a consequence of this proliferation, the embryo is termed conceptus (EEMs + embryo proper) and it progresses from spherical through ovoid, tubular and filamentous stages reaching a length of ~30 cm in cattle (3) and ~100 cm in pigs (57) by the time of implantation, which starts about D14 in pigs (58), D15 in the sheep (59), and D19 in cattle (60). In other non-ungulate domestic species, such as rabbits and horses, the blastocyst also experiences a massive growth of EEMs before implantation, reaching up to 20 mm in horses and 5 mm in rabbits, but it remains spherical (8).

Besides the massive proliferation of EEMs, major developmental events take place in the epiblast before implantation: an anterioposterior axis is established that will outline the body plan, and the three germ layers become specified, together with the germline (61–63). Before the RL disintegrates, the epiblast forms a small cavity that will be opened once the RL disappears, unfolding the epiblast (64, 65). This contrasts with murine and human development, where the epiblast cavitates to form the amniotic cavity (66–68) (Figure 1). Roughly concomitant to RL disintegration, the ungulate epiblast develops into a clearly identifiable circular light structure: the embryonic disc (ED), where epiblast cells develop tight junctions and form a basal lamina toward the hypoblast (63). When the ED is fully formed, expression of core pluripotency markers SOX2, OCT4, and NANOG is restricted to the epiblast (21, 25, 69). During the next days, the ED will acquire an oval shape and a higher density at the posterior edge, associated with the ingression of the first cells into the primitive streak and the beginning of gastrulation. Some cells at the posterior part of the epiblast start to express the mesoderm marker *BRACHYURY* (T) and to downregulate *SOX2* before the primitive streak is morphologically visible (59, 69–72). These T-positive cells will be the first cells to egress into the space between the epiblast and the hypoblast and will form the mesoderm, which quickly migrates to cover the whole embryonic disc. At the same time, more epiblast cells continue to egress through the primitive streak to form the endoderm, which lies on the dorsal hypoblast, while the mesoderm forms a mesenchyme between the epiblast and the endoderm. Epiblast cells that do not pass through the streak will form the ectoderm (73, 74). The primitive streak will be extended in an anterior direction, being the anterioposterior axis of the embryo proper aligned with the proliferation of the extra-embryonic membranes (i.e., conceptus elongation axis) (75).

Our knowledge of the genes and signaling pathways controlling gastrulation in mammals is mainly derived from the mouse embryo, in which gastrulation occurs following implantation. Key genes involved in gastrulation such as *BRACHYURY, EOMES, BMP4, NODAL, CER1*, or *FOXA2* seem to play conserved roles in ungulates, although with some differences in their location and temporal expression (3, 59, 63, 65, 71, 76, 77).

## Epiblast Development Constitutes the Major Obstacle for Embryo Survival

The developmental defects ultimately leading to embryo mortality during conceptus elongation have been difficult to explore, given the challenges for obtaining elongating embryos *in vivo*. However, several *in vitro* evidences and *in vivo* observations point to the development of the epiblast as the most vulnerable process. *In vitro* evidences show that the requirements for trophectoderm and hypoblast development are less restrictive than those required for epiblast survival. Primary bovine trophectoderm cell cultures can be established using relatively simple media supplemented with 10 % serum (78, 79), whereas conditions required for truly pluripotent epiblast cell culture in farm animals remain to be captured (80). In the same line, early *in vitro* culture systems designed for the development of post-hatching ungulate embryos were successful in achieving trophectoderm proliferation and some degree of hypoblast migration, but the epiblast degenerated (81–83). To attain epiblast survival *in vitro*, we required a way more complex medium (termed N2B27) containing aminoacids, lipids, vitamins, hormones, and growth factors not present in previous systems (7). Yet, under our system epiblast survival is observed in 55–60% of the *in vitro* produced embryos, whereas trophoblast and hypoblast proliferation is found in all surviving structures.

Failure in epiblast development has been also observed *in vivo*. Embryo transfer of *in vitro* produced (IVP) embryos often results in lower pregnancy rates compared to their *in vivo* counterparts, as it will be discussed below, and failures in epiblast development seem to be the main responsible for such developmental arrest. IVP-derived bovine conceptuses have been reported to exhibit smaller EDs than their *in vivo* counterparts (84), and multiple studies have reported a remarkably high percentage (23–65%) of IVP-derived conceptuses lacking EDs (4, 85–90), as reviewed by Ealy et al. (91). Impaired ED development has also been observed in ovine and bovine embryos produced by somatic cell nuclear transfer (SCNT), a technology that induces pleiotropic effects over different lineages (92, 93). Interestingly, embryos lacking an ED were also observed after SCNT at a very high rate, ranging from 20 to 58% (Table 2) (89, 94, 96–99), and failures in a mechanism that populates inner cells based on asymmetric divisions of outer cells have been proposed to be responsible for developmental arrest in SCNT rabbit embryos (95). Moreover, transcriptional alterations in embryonic lineages of SCNT embryos were 10–20-fold more abundant in the epiblast than in extraembryonic lineages, both in cattle and mice (89, 98, 101, 102). Accordingly, some authors have observed that normal elongation in SCNT cattle embryos was more frequent (46/50 embryos) than normal ED formation and gastrulation (38/50) (98).

**Table 2:** Embryonic disc development in embryos produced by assisted reproductive technologies.

**Species**	**ART**	**Embryo transfer**	**Embryo recovery**	**ED rate (%)**	**References**
Sheep	AI	D6	D11	5/6 (83)	(94)
	AI+IVC	D6 (vit)	D11	6/6 (100)	
	SCNT	D6 (vit)	D11	7/13 (54)	
	AI	D6	D13	9/9 (100)	
	AI+IVC	D6 (vit)	D13	6/6 (100)	
	SCNT	D6 (vit)	D13	9/13 (69)	
Cow	AI	D7	D16	7/19 (37)	(84)
	IVF	D7	D16	6/17 (35)	
Cow	AI	D6–D7	D14	18/20 (90)	(96)
	IVF	D6	D14	13/18 (72)	
	SCNT	D6	D14	24/33 (73)	
Cow	IVF	D7 (vit+fresh)	D14	11/20 (55)	(89)
	SCNT	D7 (vit+fresh)	D14	8/19 (42)	
Cow	IVF	D7	D12	–/227 (68)	(4)
	IVF	D7	D13	–/69 (78)	
	IVF	D7	D14	–/182 (83)	
Cow	IVF	D7	D17	5/6 (83)	(97)
	SCNT	D7	D17	12/19 (63)	
Cow	IVF	D7	D14–D15	19/20 (95)	(99)
	SCNT	D7	D14–D15	34/46 (74)	
Cow	IVF	D7	D15	6/7 (83)	(85)
Cow	AI	–	D18	10/10 (100)	(98)
	IVF	D7	D18	10/10 (100)	
	SCNT	D7	D18	24/30 (80)	
Cow	IVF	D7	D13–D14	15/20 (75)	(100)
	SCNT (transgenic cells)	D7	D13–D14	9/12 (75)	
Cow	IVF	D7	D14	20/26 (79)	(90)

*AI, Artificial Insemination; IVC, In vitro culture; SCNT, Somatic Cell Nuclear Transfer; “vit”, vitrified/frozen*.

## Lineages Specification Markers to Assess Embryo Quality in Farm Animals

*In vitro* embryo production enables a myriad of applications in livestock species, ranging from boosting the number of embryos obtained from females of high genetic merit to overcoming infertility problems associated to heat stress (103–105). According to the International Embryo Transfer Society (IETS), while the number of *in vivo* derived embryos transferred seems to have stabilized in cattle, the use of *in vitro*-produced (IVP) embryos is currently increasing (742,908 embryos transferred worldwide in 2018) (106). However, despite all efforts performed to optimize assisted reproductive technologies (ARTs), embryo production systems are still not fully efficient and important differences have been reported between *in vitro* and *in vivo* embryos (107). Many studies have shown that pregnancy rates after transfer of an *in vitro* embryo are between 10 and 40% lower than with embryos generated by artificial insemination or by Multiple Ovulation Embryo Transfer (MOET) (6, 108–111). As previously mentioned, IVP embryos often show compromised development of embryonic lineages—particularly the epiblast—following embryo transfer, and it has been estimated that 80% of pregnancy failures following embryo transfer of IVP embryos occur before day 40 of pregnancy (91). Unfortunately, these rates have not improved in the last decades.

In order to improve *in vitro* embryo production techniques, it is essential to assess embryo quality, i.e., the odds of post-transfer survival, to determine which modifications of current protocols are beneficial for subsequent embryo survival. Arguably, the best embryo quality assessment would be the analysis of embryo development following embryo transfer, but this test holds two major drawbacks (1) it is expensive, time-consuming and requires the use of experimental animals and (2) it is inherently bound to intrinsic variabilities in uterine receptivity between females (112, 113). Morphological evaluation (114), widely used both in humans and farm animals to select embryos before transfer due to its non-invasive nature, is certainly useful, as pregnancy rates are higher when better-quality grade embryos are transferred (115–118). However, embryo grade is a subjective criterion; it does not always reflect competence to establish pregnancy (119), and it does not necessarily infer proper development of embryonic lineages. For instance, early mouse mutant embryos lacking a specific cell lineage cannot be visually distinguished from their wildtype counterparts, although they hardly progress beyond implantation (37, 120–123). In this perspective, the analysis of the development of specific lineages provides deeper insights of embryo quality.

Successful development of the first cell lineages is essential for implantation and further development to term (124). The most commonly used method to analyse the first lineage differentiation (i.e., ICM vs. TE) in blastocysts from livestock species has been differential cell staining, a technique based on selective permeabilization of the outer blastocyst cells which will be subsequently stained with propidium iodide (125, 126). Unfortunately, this technique only provides information on cell location, but fails to assess if those cells are properly committed to TE or ICM, as the expression of specific lineage markers is not determined. Recent studies performing single-cell transcriptomics in farms animals have enabled the identification of lineage markers. The first study using this technology in mouse pre-implantation embryos (127) was soon followed by other reports in human (24, 48), monkey (49), cow (18, 19, 128) and pig (21), which revealed relevant differences between rodents and non-rodents mammals. In bovine, two studies based on single cell qPCR analysis of IVP morulae and expanded blastocysts observed that while some classical hypoblast markers in the mouse (*GATA6, GSC*, and *HNF4A*) were not specific to this lineage in bovine, *SOX17, GATA4*, and *PDGFRA* were largely specific (18, 19). Furthermore, the core pluripotency markers *NANOG, SOX2*, and *OCT4* were detected in epiblast cells, although *NANOG, FGF4*, and *TDGF1* were deemed as the most epiblast-specific, and trophectoderm cells exclusively expressed *CDX2, GATA2, GATA3, KRT8, PECAM1*, or *DAB2* (18, 19). In the pig, scRNAseq of *in vivo-*derived morulae, early and late blastocysts, and spherical embryos, revealed that although *NANOG, SOX2*, and *OCT4* are expressed in epiblast cells, *SOX2* is the most specific epiblast marker, followed by *NANOG* (21). *GATA2* and *GATA3* were reliable trophoblast markers, while *CDX2* was barely expressed in early blastocysts, and *TEAD4* was expressed in all cell lineages. Finally, hypoblast cells were characterized by specific expression of *SOX17, PDGFRA, GATA4*, or *NID2* (21).

**Figure 2:**
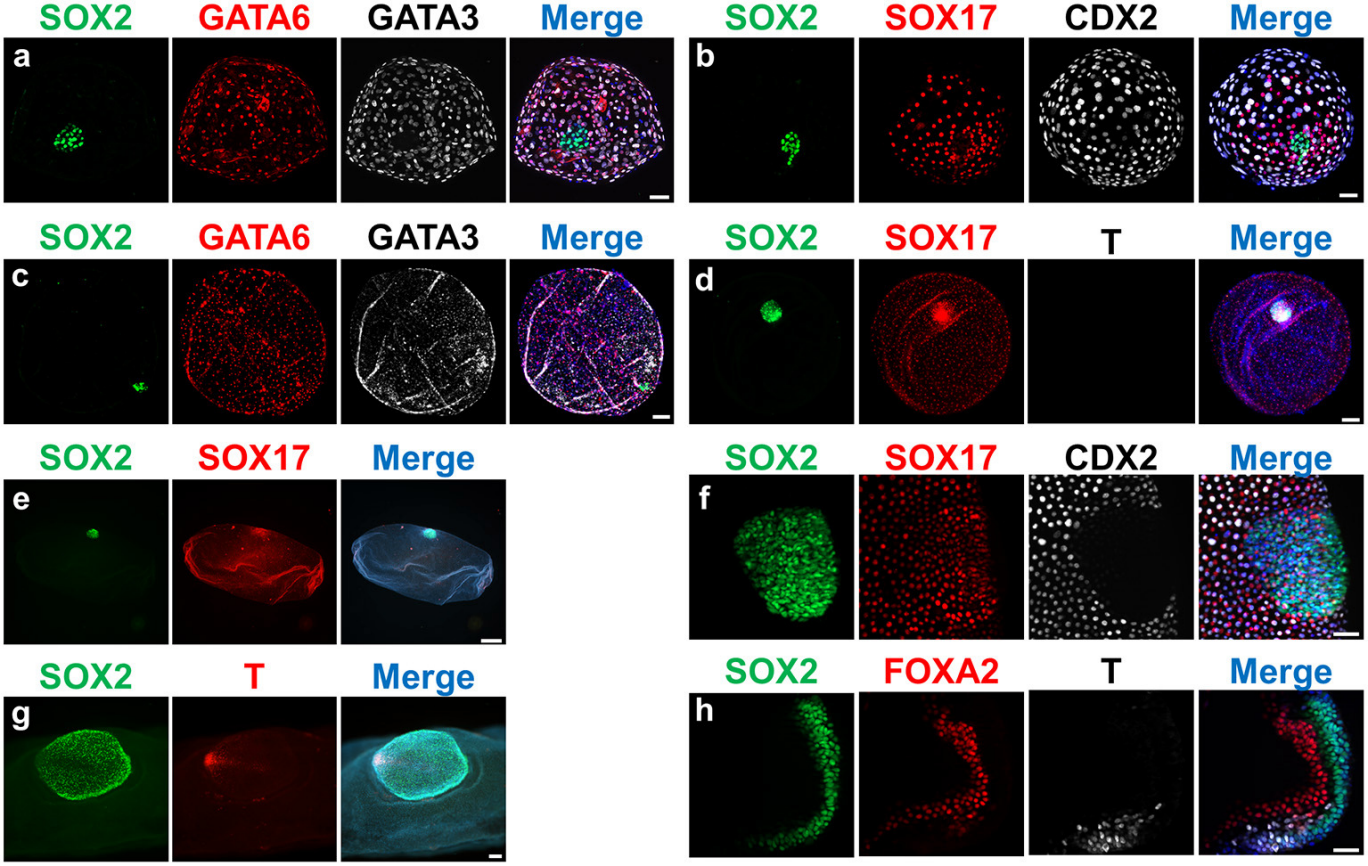
Expression of lineage-specific markers in ovine embryos at different developmental stages. Fluorescence images of embryos stained for SOX2 (epiblast); SOX17/GATA6/FOXA2 (hypoblast/endoderm); CDX2/GATA3 (trophectoderm); T (mesoderm). Nuclei were counterstained with DAPI (merge). **(a,b)** D9 Hatched blastocysts; **(c,d)**: D11 Spherical embryos; **(e)** D13 Tubular embryo; **(f)** D13 ED without Rauber's layer; **(g)** gastrulating ED; **(h)** section of a gastrulating ED. Scale bars = 50 μm for **(a,b,f,h)**; 100 μm for **(c,d,g)**; 500 μm for panel **(e)**.

**Figure 3:**
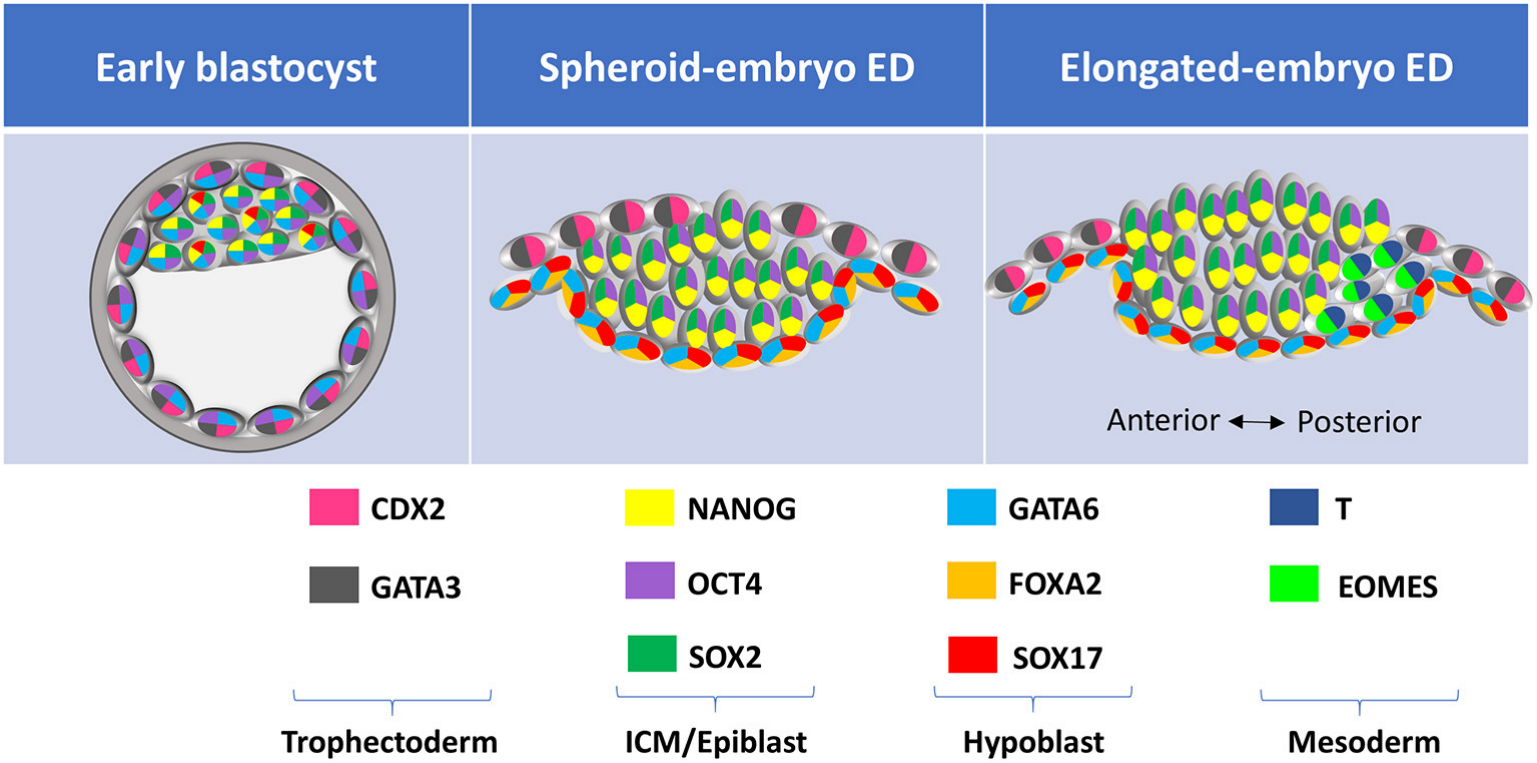
Lineage-specific markers expressed at different developmental stages in farm ungulate embryos. ED, embryonic disc; ICM, inner cell mass; TE, trophectoderm.

Expression of different lineage-specific markers can be analyzed at the protein level through embryo immunostaining (Figure 2). Although most commercial antibodies are designed to react with mouse and human proteins, some of them can also be used to label pig, sheep and cow embryos (Figure 3 and Table 3). Antibodies against the core pluripotency markers OCT4, NANOG, and SOX2 have been regularly used to label ICM and epiblast cells. Particular caution must be paid when analyzing OCT4, as this protein is expressed in all cell lineages in bovine and porcine blastocysts (25, 26, 130, 133), being its expression restricted to the epiblast only at later stages [E11 in bovine (25)]. NANOG protein has been specifically detected in ICM cells in bovine (26, 51, 131, 134) and porcine blastocysts (21). However, SOX2 seems to be the most specific epiblast marker in pig (21, 55, 130, 135), sheep (Figure 2) and bovine embryos (7, 30, 31, 131, 134). Regarding TE markers, CDX2 remains the most commonly used marker, being TE-specific in pig (26, 130), sheep (Figure 2) and cow blastocysts (7, 25, 26). In our experience (unpublished observations, Figure 2) GATA3 is also a reliable TE marker in porcine, ovine and bovine embryos. Finally, regarding to hypoblast, SOX17 specifically labels hypoblast cells from the blastocyst up to the elongated conceptus in bovine (7), ovine (Figure 2) and porcine embryos (21, 69). Other classical hypoblast markers in mice include GATA6, ubiquitously expressed in early blastocysts and becoming restricted to hypoblast cells in late bovine (26, 51), ovine (Figure 2) and porcine blastocysts (130), and GATA4, ubiquitously expressed in bovine late blastocysts and becoming specifically restricted to hypoblast cells in post-hatching E10.5 embryos (51, 129), and in some cells close to the epiblast in late pig blastocysts (132).

**Table 3:** Lineages-specific antibodies that label ungulate embryos.

**Lineage**	**Marker**	**Species**	**Antibody**	**Reference**
Inner cell mass/epiblast	OCT4	Bovine	Abcam, ab-19857	Own unpublished observations
			Santa Cruz, sc-8628^*^	(31)
			Santa Cruz, sc-9081^*^	(25)
		Porcine	Abcam, ab-27985	(129)
			Santa Cruz, sc-5279	(26)
			Santa Cruz, sc-8626^*^	(31, 55, 130)
	NANOG	Bovine	Abcam, ab-21603	(26)
			eBioscience 14-5768	(131)
		Porcine	Peprotech 500-P236	(21, 129, 132)
			Abnova, PAB6837	(130)
	SOX2	Bovine	R&D, AF2018	(131)
			Millipore, AB5603	(30)
			Biogenex, AN833-RTU	(31)
		Bovine, porcine	Santa Cruz, sc-17320^*^	(30, 130)
		Bovine, ovine, porcine	Invitrogen, 14-9811	(7) and own unpublished observations (Figure 2)
		Porcine	R&D, MAB2018	(129)
			Santa Cruz, sc-17320^*^	(55)
Trophectoderm	CDX2	Bovine	Abcam, ab-74339	(25)
		Bovine	Abcam, ab-7848	(31)
		Porcine, bovine	Chemicon, AB4123	(26)
		Bovine, ovine, porcine	Biogenex, MU392A-UC	(7, 130) and own unpublished observations (Figure 2)
	GATA3	Bovine, ovine, porcine	Abcam, ab-199428	Own unpublished observations (Figure 2)
Hypoblast/definitive endoderm	GATA6	Bovine	Santa Cruz, sc-9055^*^	(26)
		Bovine, ovine	R&D, AF1700	Own unpublished observations (Figure 2)
		Porcine	Abcam, Ab22600	(130)
	GATA4	Porcine	Santa Cruz, sc-25310	(129)
			Santa Cruz, sc-1237^*^	(132)
	SOX17	Bovine, ovine, porcine	R&D, AF1924	(7, 21, 69) and own unpublished observations (Figure 2)
	FOXA2	Bovine, ovine, porcine	Cell Signaling Technology, 8186S	(69) and own unpublished observations (Figure 2)
Mesoderm	T	Bovine, ovine, porcine	R&D, AF2085	(69) and own unpublished observations (Figure 2)
	EOMES	Porcine, ovine	R&D, MAB6166	(Own unpublished observations)

* *These antibodies have been discontinued*.

Similarly to the conventional quality assessment in blastocysts, the developmental analysis of elongated conceptuses has been traditionally based on morphology, generally limited to conceptus length (136–139). Conceptus length constitutes a good proxy for the development of extra-embryonic membranes but such development may not be coupled to the development of the epiblast, the most sensitive lineage. In other words, the “bigger is better” concept routinely applied to assess conceptus development may be wrong, as an structure only composed by EEMs (i.e., lacking an ED) will not develop any fetus. In this regard, verifying the presence and proper development of the ED is essential to determine conceptus quality. To this aim, gastrulation markers have still not received as much attention as early lineages differentiation markers, since conceptuses at gastrulation stages are less accessible for experimental studies. However, BRACHYURY protein has been first observed in nascent mesoderm cells in the posterior epiblast of ovoid pig embryos (69, 71), and both BRACHYURY and EOMES are located in the posterior part of the ED at the same time that SOX2 expression is restricted to the anterior part in elongating sheep and cow embryos (Figure 2). Finally, SOX17 and FOXA2 are expressed in the migrated hypoblast and in the definitive endoderm in pig (71), sheep and cow elongating embryos (own unpublished observations, Figure 2).

## Post-Hatching *In vitro* Development to Infer Embryo Quality of IVP Embryos

Direct assessment of the developmental potential of IVP embryos beyond the blastocyst stage (i.e., during the most vulnerable period) has been traditionally hampered by the requirement of time and resource consuming *in vivo* experiments involving embryo transfer and posterior recovery (4, 89, 100, 138), as no *in vitro* system able to support embryo development beyond blastocyst hatching was available for any farm animal. In the last years, an increasing interest has been placed on developing post-blastocyst *in vitro* culture systems to better investigate embryo development and mortality during this developmental window in different mammalian species. Human embryos have been cultured in the absence of maternal tissues up to D13 (140, 141) in a system that also allows mouse post-blastocyst culture up to egg cylinders (142). This system was further improved to allow development of human embryos up to gastrulating stages, and deep embryo characterization by single-cell transcriptome and methylome mapping (143, 144).

In farm animals, hatched blastocysts attach to the bottom of the culture dish or grow in rounded form until they collapse under normal pre-hatching embryo culture conditions. Although explanted EDs were cultured *in vitro* in rabbits (145, 146), a livestock species where gastrulation also occurs in a flat embryonic disc, limited success was achieved in ungulates until recently. Pioneer studies established an *in vitro* post-hatching development (PHD) system based on agarose gel tunnels and serum- and glucose-enriched medium that achieved some expansion of the trophectoderm and certain proliferation of hypoblast cells in bovine embryos up to D15, but hypoblast migration was incomplete and epiblast cells were unable to survive (81–83). In order to promote the development of the epiblast—the most stringent cell lineage—we have developed a system based on N2B27 medium, a defined medium composed by Neurobasal and DMEM/F12 media, and N2 and B27 supplements which was initially designed to culture neurons (147), and it was employed later for embryonic stem cells derivation and culture (148). Under this system, bovine (7) and ovine (own unpublished data) blastocysts develop beyond hatching, attaining complete hypoblast migration and epiblast survival and development into an early ED.

This pioneer system paves the way for future research focused on improving the conditions of *in vitro* embryo production or associated techniques such as embryo freezing or vitrification, as it allows direct embryo quality assessment without the need of experimental animals. The potential roles on lineages development of specific metabolites, hormones, or growth factors whose levels are altered in detrimental conditions for embryo survival, such as negative energy balance in post-partum dairy cows (90), can also be analyzed in this system.

## Conclusions and Future Directions

Being the most vulnerable period for embryo survival in ungulate species, conceptus elongation still constitutes a black box in Developmental Biology. Luckily, recent findings based on transcriptome analysis and gene ablation have started to shed light into the cell differentiation, proliferation and migration processes governing conceptus elongation, which in some cases differ greatly from those occurring in mice, the most studied mammalian model. The molecular markers of lineage differentiation in ungulates unveiled by these experiments are extremely useful to assess proper lineage development in *in vitro* produced embryos, where epiblast development has been highlighted as the major obstacle to attain a successful pregnancy. Besides, recent advances on *in vitro* culture have moved forward the limits of *in vitro* embryo development, providing a system to directly evaluate the developmental potential of IVP embryos during the most sensitive period for developmental failure. Such system provides a direct embryo quality assessment for testing diverse modifications in the IVP protocol or in associated techniques such as vitrification or freezing, aimed to improve pregnancy rates following embryo transfer.

## Data Availability Statement

The original contributions presented in the study are included in the article/supplementary material, further inquiries can be directed to the corresponding author/s.

## Author Contributions

All authors listed have made a substantial, direct and intellectual contribution to the work, and approved it for publication.

## Conflict of Interest

The authors declare that the research was conducted in the absence of any commercial or financial relationships that could be construed as a potential conflict of interest.
